# A double-blind, placebo-controlled randomised trial evaluating the effect of a polyphenol-rich whole food supplement on PSA progression in men with prostate cancer—the UK NCRN Pomi-T study

**DOI:** 10.1038/pcan.2014.6

**Published:** 2014-03-11

**Authors:** R Thomas, M Williams, H Sharma, A Chaudry, P Bellamy

**Affiliations:** 1 The Primrose Research Unit, Bedford Hospital, Bedford, UK; 2 Department of Oncology, Addenbrooke's Cambridge University NHS Trust, Cambridge, UK; 3 Department of Postgraduate Medicine, Cranfield University, Cranfield, UK

**Keywords:** nutrition, active surveillance, watchful waiting

## Abstract

**Background:**

Polyphenol-rich foods such as pomegranate, green tea, broccoli and turmeric have demonstrated anti-neoplastic effects in laboratory models involving angiogenesis, apoptosis and proliferation. Although some have been investigated in small, phase II studies, this combination has never been evaluated within an adequately powered randomised controlled trial.

**Methods:**

In total, 199 men, average age 74 years, with localised prostate cancer, 60% managed with primary active surveillance (AS) or 40% with watchful waiting (WW) following previous interventions, were randomised (2:1) to receive an oral capsule containing a blend of pomegranate, green tea, broccoli and turmeric, or an identical placebo for 6 months.

**Results:**

The median rise in PSA in the food supplement group (FSG) was 14.7% (95% confidence intervals (CIs) 3.4–36.7%), as opposed to 78.5% in the placebo group (PG) (95% CI 48.1–115.5%), difference 63.8% (*P*=0.0008). In all, 8.2% of men in the FSG and 27.7% in the PG opted to leave surveillance at the end of the intervention (*χ*^2^ *P*=0.014). There were no significant differences within the predetermined subgroups of age, Gleason grade, treatment category or body mass index. There were no differences in cholesterol, blood pressure, blood sugar, C-reactive protein or adverse events.

**Conclusions:**

This study found a significant short-term, favourable effect on the percentage rise in PSA in men managed with AS and WW following ingestion of this well-tolerated, specific blend of concentrated foods. Its influence on decision-making suggests that this intervention is clinically meaningful, but further trials will evaluate longer term clinical effects, and other makers of disease progression.

## Introduction

Diets deficient in polyphenols and other natural plant-based phytochemicals found in herbs, spices, fruit, teas, colourful vegetables and other healthy plant-based foods, have been linked with higher risks of cancer particularly breast,^1, 2^ pancreas,^3^ ovary,^4^ skin,^5^ prostate,^6, 7^ bowel^8^ and oesophagus.^9^

The protective benefits of polyphenols, however, do not to stop after a diagnosis of cancer. Breast cancer survivors taking higher levels of fruit and vegetables had a lower recurrence^10^ and those with a higher dietary intake of lignans, isoflavones, flavanones within soy-rich foods or green tea had a lower risk of breast cancer death.^11, 12, 13^ Individuals with skin cancer who had higher leafy, green vegetable intake had a lower rate of new cancer formation.^5^ Men adopting healthy diets after prostate cancer were shown to have slower PSA progression.^14, 15^

The potential benefits of concentrating foods into a pill has been the subject of extentive evaluation. Up to now, research has focused on supplements containing specific, extracted chemicals believed to be the anti-cancer candidates and although some studies have shown benefits,^16, 17, 18^ most have not or were actually linked to an increased risk of cancer. For example, the two vitamin A and E studies increased the risk of lung cancer^19, 20^ and an Australian study linked a similar supplement intake with more subsequent skin cancers.^5^ Long-term folate supplementation after myocardial infarct resulted in a higher cancer risk.^21^ A supplement containing vitamin C, copper and manganese did not slow PSA progression;^15^ the selenium and vitamin E cancer prevention trial (SELECT) study showed an increased prostate cancer incidence following long-term intake of vitamin E and selenium^22^ as did men in the Health Professionals Study, who took zinc.^23^ Despite some initial encouragement from cohort and small prospective studies, lycopene, saw palmetto or genistein extracts evaluated within more scientifically robust analyses did not demonstrate a benefit for either prostate cancer, benign prostatic hypertrophy or other malignancies.^1, 6, 18, 24, 25, 26^

As a consequence of these data, scientific attention has been turning towards the evaluation of concentrated polyphenol-rich whole food supplements, rather than extracted chemicals, as convenient ways to boost poor diets or further enhance already adequate diets. There are some laboratory and phase II studies to support their further evaluation and hence the rationale for this study. Men with prostate cancer, managed with active surveillance (AS) or watchful waiting (WW) for a PSA relapse after radical treatments, were selected as an ideal cohort to evaluate a lifestyle intervention as they have a useful serum marker of their disease, PSA, and medical interventions are often not indicated initially.^15^

### Rationale for the ingredients of this interventional food supplement

Pomegranate, rich in ellagic acid, has been shown in *in vitro* studies to inhibit proliferation, markers of migration, induce apoptosis and cell adhesion in breast and prostate cancer cell lines.^27^ In humans, a phase II study reported a prolongation of PSA doubling following pomegranate juice consumption and markers of oxidative stress improved.^28^ A further phase II study, gave men pomegranate seed extract with similar effects.^29^ Its influence was not felt to be via hormonal route as it affected both androgen-sensitive and -resistant human prostate cancer cells, and one of the clinical studies showed no change in testosterone levels following regular intake.^25, 30, 31, 32, 33, 34^

Green tea, rich in epigallocatechin gallate, has been shown to block ornithine decarboxylase, an enzyme which signals cells to proliferate faster and bypass apoptosis.^35, 36, 37^ It has also been reported to reduce several growth factors which promote breast and prostate cancer cell line growth, block de-differentiation and angiogenesis.^38^ Men given an extract of tea illustrated a significant reduction in the levels of several growth factors that promote cancer, as well as a beneficial effect on PSA.^35^

Broccoli, rich in isothiocyanate and its metabolite sulphoraphane, has been shown to inhibit growth and promote apoptosis in cancer cells.^39^ In humans, a study found that regular broccoli intake downregulated cancer genes linked to cancer promotion and up-regulated genes link to cancer suppression.^5, 7, 39, 40^

Curcumin, which gives turmeric its yellow colour, has been shown to slow prostate cancer cell growth, increase apoptosis, reduce markers of invasion and migration of cells.^41, 42, 43, 44^ It has been shown to inhibit tyrosine kinase activity of the epidermal growth factor receptor,^45^ have cyco-oxidase-I-mediated anti-inflammatory properties^46^ and halt the growth of stem cells that give rise to breast cancer without influencing normal breast cells.^46^

Finally, all four ingredients also have some anti-oxidant properties that are thought to protect the DNA against oxidative damage from ingested or environmental carcinogens,^36, 37, 47^ although this precise mechanism has not been confirmed clinically.^48^

The rationale and hypothesis for selecting the ingredients of this supplement were that as they originate different food sources (fruit, herb, vegetable and leaf), each with their unique profile and concentration of polyphenols, their separate anti-cancer mechanisms, summarised above, could be synergistic^49, 50^ yet at the same time their variable composition would avoid over-consumption of one particular phytochemical.^25, 30, 32, 33, 34, 41, 42, 43, 44^ The trial committee determined the concentration of each ingredient based on the amounts safely used within previous clinical studies.^25, 30, 32, 33, 34, 41, 42, 43, 44^

## Materials and methods

This was a placebo-controlled, double-blind, randomised trial designed with the aim of establishing whether supplementing the diet with a polyphenol-rich, whole food supplement containing a blend of green tea, pomegranate, broccoli and curcumin (turmeric) influenced the rate of PSA progression, compared with placebo among men with prostate cancer either managed with primary AS or WW, following a PSA relapse post-radical treatments. The 203 participants originated from across the UK and were all consented and randomised at The Primrose Oncology Unit, Bedford Hospital, from a total of 208 reviewed for eligibility, between November 2011 and July 2012. The intervention consisted of a tablet taken three times a day containing:

- broccoli powder (*Brassica oleracea*) 100 mg,
- turmeric powder (*Curcuma longa*) 100 mg,
- pomegranate whole fruit powder (*Punica granatum*) 100 mg,
- green tea 5:1 extract (*Camellia sinensis*) 20 mg equivalent to 100 mg of green tea and
- bulking agent (di-calcium phosphate), anti-caking agents (modified maize-based starch, maltodextrin and magnesium stearate) removed post trial.

The control group took a placebo containing identical bulking and anti-caking agents with 10 mg of watercress extract to provide an identical colour and substance.

All participants were men with an average age of 74 years (range 53–89 years), with histologically confirmed prostate cancer, 121 (60%) were being managed with AS, and 78 (40%) managed with WW following previous radical interventions and radical local salvage therapies had been excluded (primary radiotherapy, 65; surgery followed by radiotherapy, 8; and brachytherapy, 9). The baseline characteristics are summarised in Table 1.

**Figure 1:**
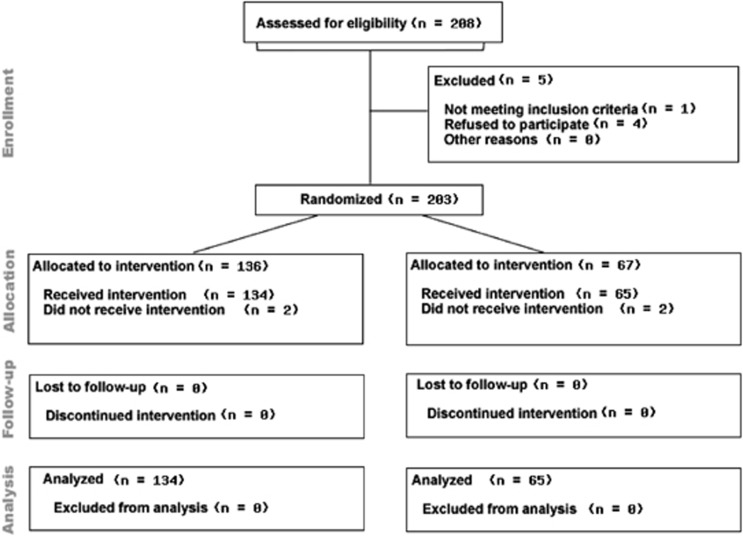
Consort diagram highlighting the flow of patients through the National Cancer Research Network Pomi-T study.

Following written informed consent, men were randomised by externally generated, numerically sequenced, opaque, tamper-proof envelopes to the food supplement group (FSG) or placebo group (PG). There was a 2:1 randomisation which resulted in 136 in the FSG and 67 in the PG. Four men withdrew consent after initial randomisation and proceeded to intervention before the 3-month consultation (two from each group), had no further relevant PSA and as such could not be included in an intention to treat analysis (Figure 1). At baseline, 3 months and 6 months post intervention, PSA, full blood count, urea and electrolytes, liver function profile, blood glucose, fasting cholesterol, C-reactive protein, body weight, height and blood pressure were measured. Adverse and favourable events were recorded in the Case Report Form according to the National Cancer Institute (NCI) common toxicity grading scale.

### Certification and quality assurance

This trial was approved by the National Ethics Committee, was peer reviewed by the National Cancer Research Institute (NCRI) Complementary Therapies Research Committee and formally adopted by the National Cancer Research Network (NCRN). The Medicines and Health Regulatory Agency (MHRA) confirmed that no MHRA licence was required as the intervention was not classified as a medicinal product. The randomisation process was outsourced and the trial methodology, collection and storage of data were verified and independently audited by an external agency to ensure adherence to European Good Clinical Practice. At the end of the trial, data were externally audited for a second time to ensure that there were no data inconsistencies or deviation from the trial design, before the database was sealed and sent for blinded analysis by the statistician at Cranfield University. The UK manufacturers of the food supplement (Power Health Products, York, UK) adhered to good manufacturing practice guidelines and performed in-house analysis for authenticity and purity (the manufacturing analysis certificates were presented to the reviewers of the publication). The food supplement and placebo tablets were supplied to the trials unit in tamper-proof, sealed containers. A batch of the supplement has been securely stored by the trust secretary and can be sent to any regulatory body at request.

### Statistical considerations

The patient representatives on the NCRI Development Committee felt that a 2:1 randomisation would be more acceptable to participants. This appeared to be a correct assertion because all but five eligible men invited to enter this study agreed to be randomised. The PSA value used for the final analysis was pre-determined to be the 6-month value or the 3-month value in the men who withdraw at this stage.

### Statistical methods

The percentage change in PSA from baseline to final measurement was evaluated using an analysis of covariance (ANCOVA) which assessed the effect of the FSG versus PG, as well as the predetermined subgroups of Gleason grade, body mass index and treatment category. Age was included as a covariate to adjust for any differences in age between groups. The interactions between the effect of the FS and each of the other categorical effects were also included in the analysis. To satisfy the assumptions of this analysis, the change in PSA had to be transformed to logarithms, *P*-values given in this section followed by ANCOVA are from the F-value in this ANCOVA. All median values were back transformed from this ANCOVA and therefore allow for the effect of any differences in other subgroups or age.

The analysis of the number of men at the end of the trial with the same or lower PSA was analysed using a χ^2^ test with 1 degree of freedom. The differences in toxicity measures between the two groups were tested using an appropriate *t*-test (that is, with equal or unequal variances), and transforming the measure if required to satisfy the assumptions of a *t*-test.

## Results

### Primary end point

In the FSG, the mean PSA rose from 6.50 to 6.81 ug l^−6^ from baseline to the end of the intervention, a median PSA percentage rise of 14.7% (95% confidence interval (CI) −3.4% to 36.7%). In the PG, the mean PSA increased from 6.50 to 10.98 ug l^−1^, a median percentage rise of 78.5% (95% CI 48.1–115.5%). The median percentage PSA increased at a significantly slower rate in the FSG group compared with the PG (difference 63.8% ANCOVA, *P*=0.0008).

### Secondary end point

The number of men with a PSA lower or the same value at trial completion was 61 (46%) in the FSG as opposed to 9 (14%) in the PG. This difference was statistically significant (χ^2^ value with 1 degree of freedom=19.58, *P*=0.000010).

### Decisions to remain on AS or WW

Twenty-five men opted to leave AS or WW at 3 months (11 in the FSG and 14 PG), and 4 after 6 months, 11 of 134 in the FSG (8.2%) and 18 of 65 (27.7%) in the PG (this difference of 19.5% was significant χ^2^ value with 1 degree of freedom, *P*=0.014). The reasons for opting out were multifactorial and at the discretion of the physician and patient who were both blind to the intervention arm but all had a rising PSA.

### Predetermined subgroup analysis

**Figure 2:**
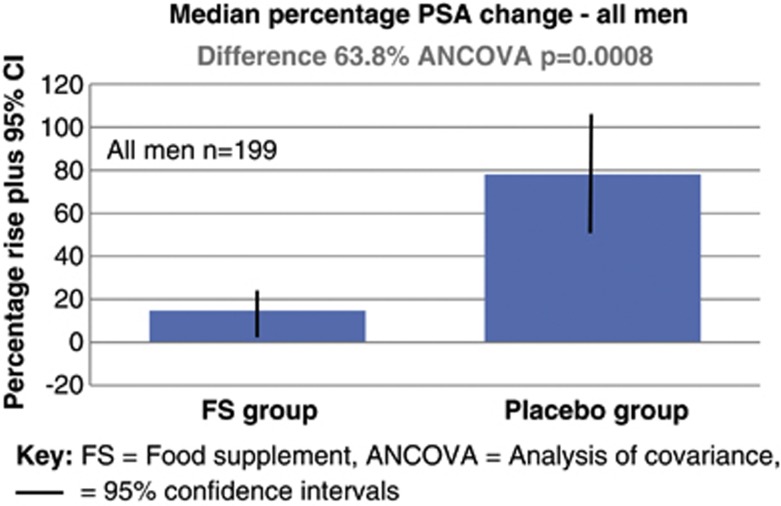
Median percentage rise in PSA between men taking the food supplement versus placebo.

**Figure 3:**
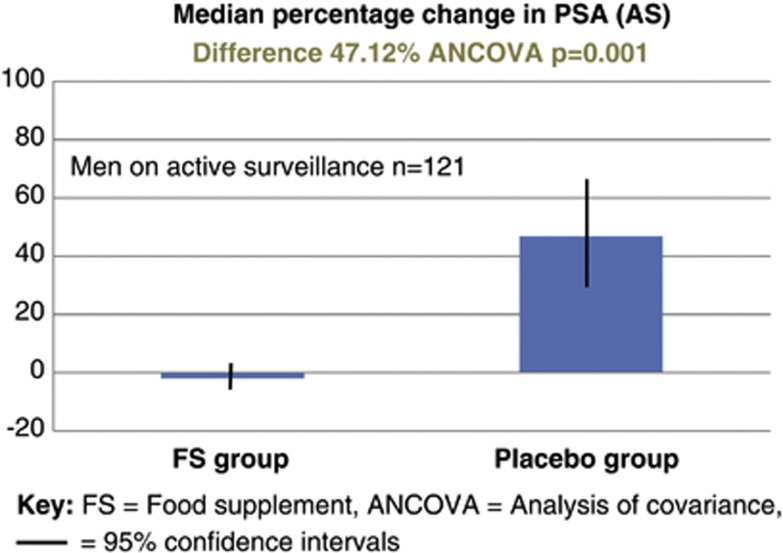
Subgroup analysis: median percentage change in PSA for the 121 men managed with active surveillance (AS).

**Figure 4:**
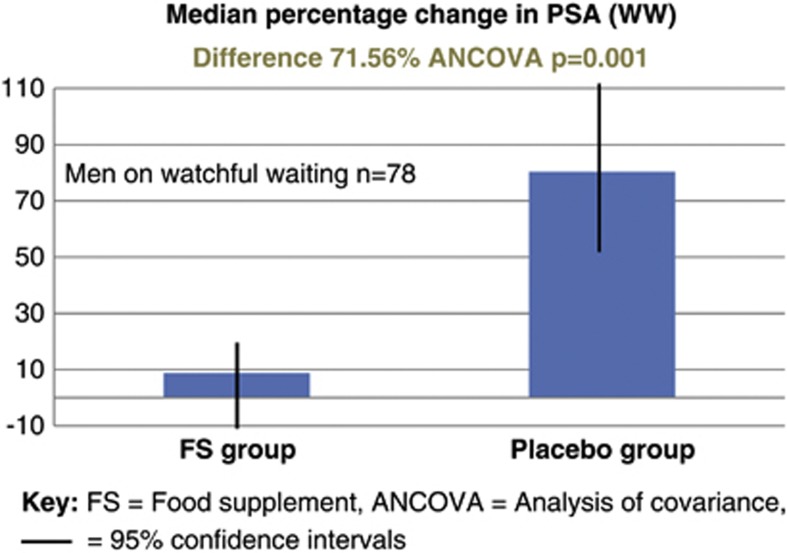
Subgroup analysis: median percentage change in PSA for the 78 men managed with watchful watching (WW; PSA relapse following previous radiotherapy).

A separate analysis of the cohort of men managed with AS (*n*=121) revealed that in the FSG the mean PSA dropped by 0.14% (95% CI −7.57 to 7.95), whereas in the PG it rose by 46.98% (95% CI 28.51–68.31); difference 47.12% (ANVOCA, *P*=0.001), see Figure 2. A separate analysis of the cohort of men managed with WW (*n*=78) revealed that in the FSG the mean PSA rose by 8.78% (95% CI −6.32 to 26.62), whereas in the PG it rose by 80.34% (95% CI 50.54–116.55); difference 71.56% (ANVOCA, *P*=0.001), see Figure 3. This difference between the median percentage change in PSA in either the AS or WW cohorts was not statistically significant (*P*=0.805 ANCOVA) (Figure 4).

There was no statistical difference between the median percentage PSA rise between men in the FSG or the PG as to whether they were overweight (⩾25 kg m^−2^) or not (*P*=0.564 ANCOVA). Although men with higher Gleason grade for the entire cohort tended to progress at a faster rate, there was no difference between the median percentage change in PSA between men in the FSG or the PG whichever Gleason grade category they were (*P*=0.089 ANCOVA). There was a significant effect of age on the median percentage change in PSA for the entire cohort with older men tending to progress at a slower rate (*P*=0.0272 ANCOVA; slope of log(MPC-PSA)=0.0098, *P*=0.0272). As there were more older men randomised to the PG, age was included as a covariate in the analysis so that the difference between the median percentage change in PSA for FSG and PG was adjusted to the mean age of the whole cohort, so that any effect on PSA between FSG and PG would have been removed.

### Other measures

There were no significant differences at the beginning or the end of the study between groups for cholesterol, blood pressure, serum glucose or C-reactive protein. Compliance, measured by counting remaining tablets in returned pots, was excellent and similar at 98.4% in the PG and 96.5% in the FSG. Sex hormones were not a predetermined analysis, as the ingredients were specifically chosen not to have phytoestrogenic properties but were measured in 64 men who had been taking the supplement for at least 3 months or more. Three of these men (5.5%) had testosterone levels below our laboratory normal range. The average testosterone (13.4 nmol l^−1^) was within the normal range as were the other sex hormones: follicle stimulating hormone, 9.2 iu l^−1^; luteinizing hormone, 7.4 iu l^−1^; sex hormone binding globulin, 41.4 nmol l^−1^; and free androgen index, 24.3%. Magnetic resonance images (MRI) of the prostate were also not included at specific time points in the trial protocol but a total of 74 of the 121 men on AS, were taken as part of their routine management clinical protocol. All these scans, in addition to their original report, were scrutinised within our multidisciplinary team meetings. Twelve (16%) men had radiologically progressive disease and in these men the average PSA rose from 7.65 to 8.67 ug l^−1^. Eight (11%) had radiological regression and in these the average PSA dropped from 7.2 to 4.1 ug l^−1^. No man in the FSG had radiological progression with a stable PSA but one man (1.3%) not taking the FS had radiological progression with a falling PSA. Although these figures were not subjected to statistical analysis as MRI was not a predetermined end point but they do give some reassurance that PSA change is linked to underlying disease status.

### Adverse events

There were 34 (24%) men who recorded adverse events (any NCI score) in the FSG group and 23 (34%) in the PG. These differences were not statistically significant (χ^2^ value with 1 degree of freedom=2.2, *P*=0.14). There were no grade ⩾3 toxicities, but one man in the FSG group had grade 2 diarrhoea. Gastrointestinal events, considered separately, occurred in 21 (15.5%) in the FSG group as opposed to 5 (7.5%) in the PG, but this difference was not statistically significant (χ^2^ value with 1 degree of freedom=2.24, *P*=0.11). No man in either group reported central nervous system symptoms, such as agitation, insomnia or tremors, none of the 30 men on warfarin reported any unexpected change in the international normalized ratio, nor did the 43 men taking ramipril report an unexpected change in their blood pressure.

### Positive events

There were 16 (12%) men who recorded positive events (mainly improved bowel and urinary function) in the FSG, and 3 (4.6%) in the PG. These differences were not statistically significant (*χ*^2^ value with 1 degree of freedom=2.67, *P*=0.10) (Table 2).

## Discussion

This study demonstrated a significant effect on the rate of PSA progression among men with prostate cancer, randomised to take this nutritional supplement compared with placebo. The difference was large, the patient characteristics were well-balanced and the trial had sufficient numbers to ensure adequate statistical power. However, there are some caveats with the trial design to consider and discuss.

The first caveat was the relatively short, 6 months, duration of the intervention which although enough time to detect an early difference, as men are often managed with surveillance for many years, a longer design would exclude the possibility that this effect was short lived. On the other hand, a longer study would have been beyond the funding constraints of this non-commercial study. Furthermore, a trial designed to take a potential placebo for longer would have been a less attractive option for men, so the rapid recruitment achieved in this study would have been less likely.

The second caveat was that the main end point relied on PSA measurement, without other formal indicators of disease progression including MRI or prostate biopsies which are now becoming routine in the management of men on AS. Although biopsy would have enhanced scientific competence, not all men currently consent a repeat intervention and hence this inclusion would have reduced the rate of recruitment, increased complexity. The cost of including MRI before and after the trial period would also have been prohibitive for the level of sponsorship available but fortunately, images were taken as part of their routine management in over half of the men in the AS group. It is of some reassurance, that PSA reflected underlying disease status as the percentage change in PSA was 10-fold lower in the men with disease shrinking on MRI compared with disease progression and no man taking the food supplement had disease progression with a stable PSA. All men choosing to continue to supplement after the trial period will now receive annual MRI's this data will subject to a future publication.

Another issue with the trial design was that the cohort included men both on primary AS and those experiencing a PSA relapse after radical treatments, deemed not eligible for salvage local therapies. The design committee believe that this was not a negative caveat as there were appropriate reasons for this choice. First, in our experience, this is precisely the group of men who are most interested in lifestyle strategies^15^ and a trial cohort should, where possible, reflect the wider population considering the study intervention. Both groups of men are similar as they have viable prostate cancer, and are not receiving other systemic therapies. Both groups have serum PSA being monitored as part of their routine clinical management. Previous nutritional studies have also included both groups.^15, 17, 26^ To support this rationale, in this study, there was no statistical difference in the effect on PSA between both cohorts, suggesting a similar effect of polyphenol-rich foods, regardless of whether patients had received radiotherapy or not.

Despite these caveats, although PSA has its short falls, men managed with AS or WW are greatly concerned with their serum levels and a rise is often a trigger for a change in their management.^31^ This was borne out in the trial, as 29 men opted for to leave AS or WW during or at the end of the study when their PSA rose, twice the percentage in the PG than the FSG. This difference, as well as achieving strong statistical significance, suggests a clinically meaningful effect. Notwithstanding avoidance of the inevitable toxicities of androgen deprivation therapy, with over a 10-fold difference in price between this food supplement and androgen deprivation therapy, a future trial should include a cost-effectiveness analysis in order to determine the magnitude of potential savings for healthcare providers.^15^

As well as price, other attractive features of this supplement were its tolerance and safety. There were no overall statistically significant difference in symptoms compared with placebo, although more men in the supplement group experienced non-significant bloating or diarrhoea, but almost as many reported beneficial effects including improved digestion and urinary symptoms. The trend towards better urinary symptoms is worthy of future scrutiny in the next study, especially as previous studies have linked turmeric with improved symptoms of prostatitis presumably via its anti-inflammatory properties.^41, 46^ One of the ingredients of the supplement, pomegranate, has been cited to be a weak inhibitor of cytochrome P450 but, reassuringly, the quantities used in this study resulted in no unexpected changes in blood pressure or international normalized ratio levels, which could have related to interference of the metabolism of ramipril or warfarin.

Although, these ingredients have previously demonstrated fundamental effects on cancer progression in laboratory studies including markers of angiogenesis, metastasis, adhesion and apoptotis,^25, 30, 32, 33, 34, 41, 42, 43, 44^ our next trial will explore these mechanisms in more detail building upon the translational designs instigated by previous researchers.^14^ Furthermore, although these ingredients were specifically selected not to have phytoestrogenic properties and the average sex hormone levels were reassuringly normal in the subgroup who had them measured, the next trial will also explore the physiological effects on participants in more detail, including serum testosterone and markers of oxidative stress.^2, 34^

In conclusion, this statistically valid double-blind randomised controlled trial has demonstrated a significant short-term effect on PSA and is food for thought for men living with prostate cancer, 50–70% of whom are reported to have taken ‘over-the-counter' supplements.^47^ The favourable effect on PSA progression was significant both in men on primary AS and those experiencing a PSA relapse after radiotherapy. This low cost food supplement was well-tolerated and also influenced clinically relevant decisions, as to whether to switch to interventions with more toxicity. Although these results do not prove a long-term effect, they have provided significant encouragement to design a major study with more comprehensive physiological, radiological and translational laboratory end points, in order to get a deeper understanding of the evidence of benefit and role for these complex and readily available foods.

## Figures and Tables

**Table 1:** Summary of baseline characteristic in the randomly assigned groups

*Baseline characteristic*	*FSG (134)*	*PG (65)*
Age (mean years)	71.8	76.4^a^
PSA (mean μg l^−1^)	6.5	6.5
Gleason grade⩽7	127 (95%)	57 (88%)
Gleason grade >7	7 (5%)	8 (12%)
Gleason grade mean (μg l^−1^)	6.5	6.2
BMI (mean kg m^−2^)	28.1	28.3
Cholesterol (mean mmol l^−1^)	4.87	4.72
BP (mean systolic/diastolic mm Hg)	146/83	150/82
Serum glucose (mean mmol l^−1^)	5.15	5.30
C-reactive protein (mean mg l^−1^)	1.51	1.74

Abbreviations: BMI, body mass index; BP, blood pressure; FSG, food supplement group; PG, placebo group.

The mean age in the PG was older by 4.4 years (*t*-test *P*=0.013) so age was included in the analysis of the percentage change in PSA as a covariate.

a Randomisation produced no statistical difference in the group characteristics except for age.

**Table 2:** Summary of the adverse and positive events

	*FSG (% of 134)*	*PG (% of 65)*	*Difference % (significance)*
*Adverse gastro-intestinal events*			
Loose bowels	6 (4.5%)	0 (0%)	4.5 (ns)
Diarrhoea (grade 1)	2 (1.5%)	0 (0%)	1.5 (ns)
Diarrhoea (grade 2)	2 (1.5%)	1 (1.5%)	0 (ns)
Diarrhoea (grade ⩾3)	0 (0%)	0 (0%)	0 (ns)
Constipation	2 (1.5%)	0 (0%)	1.5 (ns)
Flatulence	5 (3.6%)	0 (0%)	3.6 (ns)
Rectal bleeding	0 (0%)	1 (1.5%)	1.5 (ns)
Nausea	0 (0%)	1 (1.5%)	1.5 (ns)
Bloating	4 (3%)	2 (3%)	0 (ns)
All GI adverse events	21 (15.5%)	5 (7.5%)	8 (ns)
			
*Other adverse events*			
Gout exacerbation	2 (1.5%)	1 (1.5%)	0 (ns)
Worsening urinary flow	2 (1.5%)	2 (3%)	1.5 (ns)
Worsening renal function	2 (1.5%)	0 (0%)	1.5 (ns)
Weight loss	0 (0%)	2 (3%)	3 (ns)
Non-specific ‘feeling unwell'	2 (1.5%)	4 (6%)	4.5 (ns)
Miscellaneous unrelated	5 (3.6%)	9 (13.4%)	9.8 (ns)
All adverse events	34 (24%)	23 (34%)	10 (ns)
			
*Positive events*			
Improved erectile function	1 (0.75%)	0 (0%)	0.75 (ns)
Improve urinary flow	4 (3%)	1 (1.5%)	1.5 (ns)
Reduced prostatic discomfort	1 (0.75%)	0 (0%)	0.75 (ns)
All positive prostatic symptoms	6 (4.5%)	1 (1.5%)	3 (ns)
Improved bowel function	8 (6%)	0 (0%)	6 (ns)
Improved well being	2 (1.5%)	2 (3%)	0 (ns)
All positive event	16 (12%)	3 (4.5%)	7.5 (ns)

Abbreviation: FSG, food supplement group; GI, gastrointestinal; ns, non-significant difference; PG, placebo group.
